# Genomic and clinical features of endoplasmic reticulum stress factor in digestive system pan-cancer studies

**DOI:** 10.3389/fonc.2022.1072576

**Published:** 2023-01-09

**Authors:** Sheng Yao, Yuanquan Yu, Liyi Xu, Xiang Pan

**Affiliations:** ^1^ Department of Gastroenterology, the Second Affiliated Hospital, School of Medicine, Zhejiang University, Hangzhou, Zhejiang, China; ^2^ Department of Hepatobiliary and Pancreatic Surgery, the Second Affiliated Hospital, Zhejiang University School of Medicine, Hangzhou, Zhejiang, China; ^3^ Laboratory of Gastroenterology, Second Affiliated Hospital of Zhejiang University School of Medicine, Hangzhou, Zhejiang, China

**Keywords:** endoplasmic reticulum stress, digestive system pan-cancer, genomic feature, clinical feature, prognosis, tumor microenvironment

## Abstract

**Introduction:**

Digestive system pan-cancer is one of the lethal malignant tumors, which have the propensity for poor prognosis and difficult treatment. Endoplasmic reticulum (ER) stress has served as a pivotal role in the progression of the tumor, while the implication of ER stress on digestive system pan-cancers still needs elucidation, especially from the perspective of clinical outcome and that of genomic features.

**Methods:**

First, Among the ER STRESS factors from the REACTOME_UNFOLDED_PROTEIN_RESPONSE_UPR (113 genes) and HALLMARK_UNFOLDED_PROTEIN_RESPONSE (92 genes) terms, 153 ER STRESS regulators were identified after removing replicates. The somatic mutation data and copy number variation data of gastrointestinal pan-cancer were downloaded from The Cancer Genome Atlas (TCGA) database. Then, we explored the clinical outcome and genetic mutation of ER stress-related differentially expressed genes (DEGs) by multiple bioinformatics analysis. Subsequently, we analyzed the Spearman correlation between the drug sensitivity of 179 gastrointestinal anticancer drugs and the transcriptional expression of 153 ER stress factors in 769 cancer cell lines of the GDSC2 cohort. Next, ssGSEA method was used to quantify the immune cell infiltration scores in the tumor microenvironment, and Spearman correlation was used to calculate the correlation between ER stress scores and immune cell infiltration. Finally, we analyzed the cellular origin of ER stress factor dysregulation.

**Results:**

We analyzed the genomic changes and clinical outcomes of ER stress factors in different tumors of gastrointestinal pan-cancer. Endoplasmic reticulum stress factor (ER) in digestive tract tumors showed high SNV mutation frequency, less methylation dysregulation and was associated with multiple oncogenic pathways. Endoplasmic reticulum stress factor (ER) is a risk factor for many cancers, but the effect on overall survival in rectal adenocarcinoma is opposite to that in other gastrointestinal tumors. And ER stress factors are highly correlated with drugs that target important pathways.

**Discussion:**

Based on the clinical prognosis and genomic analysis of ER stress-related factors in patients with gastrointestinal pan-cancer, this study provides a new direction for further research on gastrointestinal pan-cancer.

## Background

Endoplasmic reticulum (ER) stress is affected by various intracellular and extracellular factors and is characterized by the accumulation of unfolded or misfolded conditions. For any cancer, various genetic, transcriptional, and metabolic abnormalities generate an unfavorable microenvironment that leads to persistent ER stress in tumor cells, ultimately affecting their function, fate, and survival (1). Three transmembrane proteins, activated transcription factor 6 (ATF6), inositol requirement enzyme 1α (IRE1α), and PrKR-like endoplasmic reticulum kinase (PERK), operate as ER stress sensors in mammalian cells. During ER stress, binding-immunoglobulin protein (BiP; also known as GRP78) dissociates from the sensor (2). This is an adaptive mechanism that subsequently induces the unfolded protein response (UPR; also known as the ER stress response), which can restore ER homeostasis through various mechanisms, including transcriptional reprogramming, mRNA decay, global translational decay, removal of misfolded proteins through the ER-associated protein degradation system, and recycling of misfolded proteins and cellular materials through the induction of autophagy (3).

The ER stress response usually promotes cell adaptation to stress and survival by restoring ER homeostasis, but unresolved or extreme ER stress will cause the death of cell. One such example is the use of proteasome inhibitors as anticancer agents, which induce unresolved fatal ER stress (4). Furthermore, specialized secretory cells with constitutive UPR activity are highly sensitive to additional ER stress, triggering cell death (5). Importantly, signaling *via* ER stress sensors is now understood to modulate UPR-independent transcriptional and metabolic pathways in a cell-specific and environment-dependent manner to regulate cellular phenotypes associated with cancer initiation, progression, and treatment response or resistance. As the ER is in close and dynamic contact with other organelles, such as the nucleus, mitochondria, and Golgi apparatus, intrinsic changes in the ER can greatly affect the regulation of entire cellular processes.

The protein folding capacity of the ER in both malignant and stromal cells is dynamically disturbed by various stressors enriched in the tumor microenvironment (TME) (6). Extreme hypoxia, nutrient availability, intracellular accumulation of reactive oxygen species and low pH are common features in the TME that perturb ER homeostasis and induce ER stress (7). In addition to adverse environmental conditions for tumorigenesis, genetic alterations in cancer cells can exacerbate ER stress and promote sustained activation of the UPR pathway, such as the UPR in oncogenic transformation and tumor growth, UPR in metastasis and dormancy, and modulation of the tumor immune microenvironment by ER stress in the cancer cell (8). ER, as a cell center, senses and integrates various intracellular changes, as well as extracellular conditions and factors (9). Indeed, the intrinsic disruption of ER homeostasis in tumor-infiltrating leukocytes is considered a key mechanism for promoting malignant progression and immune escape (10). Furthermore, in the TME, myeloid cells, T, and NK cells help to maintain the harmful ER stress response that permeates immune cells (11–13).

Ample studies have suggested that endoplasmic reticulum stress shows significant effect through tumorigenesis, development, metastasis, angiogenesis, drug resistance and so on. A study show an increased UPR in dormant malignant cells from patients and mouse models of cancer in pancreatic ductal adenocarcinoma (14). Endoplasmic reticulum stressor thapsigargin, given to mice with CT26-derived colon tumors, promoted the recruitment and immunosuppressive activity of MDSCs, which could be attenuated after treatment with a compound that alleviates protein folding stress (15). HCC cells treated with endoplasmic reticulum stress source tunicamycin release exosomes containing a large amount of miR-23a-3p, and up-regulate the expression of programmed cell death protein-1 ligand 1 (PDL1) in macrophages by regulating the PTEN-AKT pathway, and histological expression of endoplasmic reticulum stress markers BiP, ATF6, PERK and IRE1α in human HCC specimens was associated with increased infiltration of CD68+PDL1+ macrophages and poor prognosis of patients (16). However, there are few studies on the correlation between endoplasmic reticulum stress factors and overall gastrointestinal tumors. We focused on the analysis of Genomic and clinical features.

This article focuses on the potential drivers of ER stress in the TME, as well as epigenetic alterations in ER stress factors, the interplay between oncogenic events and ER stress factors, and the mechanisms by which ER stress factor pathways influence tumors, and dictate malignant progression and treatment response.

## Materials and methods

### Analysis of somatic mutations and copy number changes

Among the ER STRESS factors from the REACTOME_UNFOLDED_PROTEIN_RESPONSE_UPR (113 genes) and HALLMARK_UNFOLDED_PROTEIN_RESPONSE (92 genes) terms, 153 ER STRESS regulators were identified after removing replicates. The somatic mutation data and copy number variation data of gastrointestinal pan-cancer were downloaded from The Cancer Genome Atlas (TCGA) database. Only non-silent mutations were retained for mutation types (Frame_Shift_Del, Frame_Shift_Ins, In_Frame_Del, In_Frame_Ins, Missense_Mutantion, Nonsense_Mutation, Nonstop_Mutation, Splice_Site, Translation_Start_Site).

### mRNA expression and ER STRESS score

The clinical information and fragments per kilobase million (FPKM) of esophageal carcinoma (ESCA), stomach adenocarcinoma (STAD), liver hepatocellular carcinoma (LIHC), rectum adenocarcinoma (READ), colon adenocarcinoma (COAD), pancreatic adenocarcinoma (PAAD) were got from UCSC (https://xenabrowser.net/datapages/). The R package “limma” was used to conduct differential expression analysis, and ER STRESS factors were considered to be differentially expressed between tumor and paracancer samples when |log2FC|>1 and FDR<0.05. The average value of the expression of all ER stress regulators in each sample was used as the ER stress score for that sample. The ER stress score represents the endoplasm overall estimation of the net stress factor expression.

### Biological pathway analysis

To determine the biological pathways related to ER stress, we calculated 50 pathway scores in Msigdb Hallmark using ssGSEA, and analyzed the Spearman correlation between ER stress factors and pathway scores. Pathways with corrected P-values < 0.05 were considered to be associated with ER stress.

### Analysis of immunomodulatory and immune cells

A recent publication by the TCGA immune response working group (17) provides a list of immune modulators, which are a group of immunomodulatory genes, including antigen presenting factors, ligands, and receptors, which play crucial roles in tumor immunotherapy. The gene sets marking each TIME infiltrating immune cell type was obtained from Charoentong’s study, and the gene sets were rich in various human immune cell subtypes, including activated CD8^+^ T cells, activated dendritic cells, macrophages, natural killer T cells, and regulatory T cells (18, 19). Enrichment scores calculated by sGSEA analysis were used to represent the relative abundance of each time-infiltrated cell in each sample. For the correlation between ER stress score, immune cell infiltration score, and immunomodulators, a corrected P-value of < 0.05 was considered the threshold.

### DNA methylation analysis

TCGA database (http://gdac.broadinstitute.org/) contains 450k DNA methylation information. To assess the effect of promoter DNA methylation on ER stress factor expression, we only retained methylated sites in the TTS-1,500 bp to +500 bp range. The absolute value of Beta change between the normal and tumor samples was > 0.2 and FDR<0.05 was defined as promoter DNA methylation dysregulation. We performed Spearman correlation analysis of promoter DNA methylation dysregulated sites and ER stress factor expression and retained the minimum Spearman correlation coefficient.

### Survival analysis

The clinical information on patients with cancer was obtained from TCGA database. Patients were divided into high- and low-expression groups according to the median expression of ER stress factor. The R software packages Survival and Survminer were used to draw Kaplan–Meier SUR overall survival curves. The log-rank test was used to determine the statistical significance of survival differences between the high- and low-expression groups. Additionally, univariate Cox analysis was performed to determine the prognostic impact of each ER stress factor on patients with gastrointestinal pan-cancer.

### Drug sensitivity analysis

The IC50 of the 769 cancer cells is the standardization the gene expression patterns and 179 types of digestive tract cancer drugs were downloaded from GDSC (http://www.cancerrxgene.org/downloads). To assess the correlation between small-molecule drug sensitivity and gene expression levels, we calculated the Spearman correlations between ER stress factor expression and the IC50 of the drug. The significance threshold was *P*<0.05.

### Cellular source of ER STRESS dysfunction

Frist, we downloaded single-cell data for gastric, liver, colorectal, and pancreatic cancers were downloaded from the TISCH database, examined the normalized data and exmployed the Find VariableFeatures function to determine the top 2000 highly variable genes (variable features identified based on variance stabilization transformation (“VST”). Subsequently, the ScaleData function was used to scale all genes, and the RunPCA function was used to reduce the dimension of the top 2000 highly variable genes. To identify the cell clusters, we selected DIM = 15 and clustered the cells through the “FindNeighbors” and “FindClusters” functions (resolution = 0.2). Next, we selected the top 15 principal components to further reduce the dimensionality using the tSNE approach, before annotating the cell types using the annotation information submitted by the authors. Finally, we constructed a violin diagram to show the expression of ER stress factors in different cell types.

## Results

### Expression of ER stress factors is altered in the digestive system

**Figure 1:**
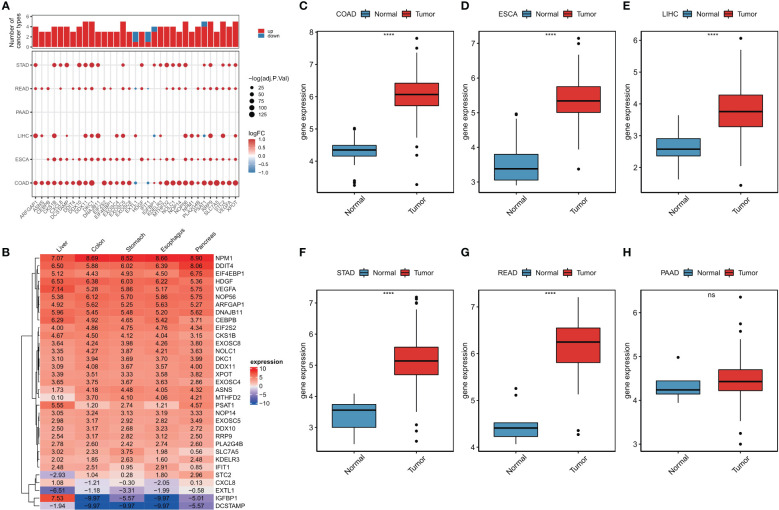
Abnormal expression of endoplasmic reticulum stress factor in digestive tract tumors. **(A)** Endoplasmic reticulum stress factors that show differential performance in at least three cancers; **(B)** The expression of ER stress factors in normal tissues that show differential expression in at least three cancers; Expression of **(C-H)** DKC1 gene in different digestive tract tumors. ****P<0.0001; ns, not significant.

A flowchart of this study is shown in **Figure S1**. Using limma analysis, we analyzed the expression of 153 ER stress factors (**Tables S1-S7**) in different tumors in digestive tract cancer; the standard for the screening of differentially expressed genes was |log2FC|>1 and FDR<0.05. The differential expression of ER stress factors is shown in **Table 1**. We selected ER stress factors that were differentially expressed in at least three cancers. The results showed that these ER stress factors were generally highly expressed in gastrointestinal pan-cancer, with more obvious differential expression in COAD, LIHC, and ESCA; among which, DDX11, DKC1, EXOSC5, NOP56, and other genes were differentially expressed in the most cancer types, as shown in **Figure 1A**. The dyskeratosis congenita 1 (DKC1) gene was discovered because of its mutation leading to dyskeratosis congenita. Hou et al. showed that DKC1 directly binds to the HIF-1α promoter region, enhances HIF-1α transcription, increases HIF-1α and VEGF expression, and promotes CRC progression. Wang et al. showed that STC2 was upregulated in hepatocellular carcinoma (HCC) and correlated with the tumor size and diversity. Abnormal expression of STC2 promoted the growth, invasion, and colony formation of cancer cells, whereas silencing STC2 delayed the cell cycle in the G0/G1 phase. Studies have shown that STC2 regulates cyclin D1 and activates ERK 1/2. Moreover, overexpression of STC2 has been observed in lung cancer cells, and STC2 knockdown has been shown to inhibit the growth, colony formation, invasion, and metastatic capacity of cancer cells. These results suggest that ER stress factors play different roles in digestive system tumors. Next, using the GTEx database, we investigated the expression of the above differentially expressed ER stress factors in different normal tissues of the digestive tract. The results showed that IGFBP1 had low expression in normal tissues, but high specific expression in LIHC, as shown in **Figure 1B**. And results show the expression of DKC1 in different digestive tract tumors in **Figures 1C–H**.

**Table 1:** The differential expression of ER stress factors.

Cancer	Up	Down
COAD	35	5
ESCA	54	0
LIHC	45	4
PAAD	0	0
READ	25	5
STAD	23	2

### Genome of ER stressors is altered in digestive tumors

**Figure 2:**
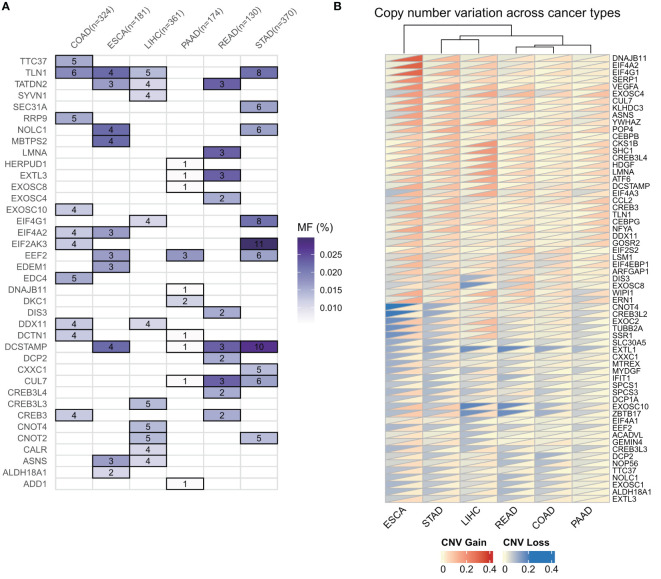
Genomic alterations of endoplasmic reticulum stress factors in gastrointestinal tumors. **(A)** Endoplasmic reticulum stress factor SNV mutation in digestive tract tumors; **(B)** CNV variation of endoplasmic reticulum stress factor in gastrointestinal tumors.

To investigate the genomic alterations of ER stress factors in gastrointestinal tumors, we first calculated the frequencies of CNV variants (amplifiers and deletions) and SNV mutations (non-silent mutations) in a pan-cancer cohort of patients with six types of cancer (**Figures 2A, B**). The results showed that the TLN1 (talin-1) gene had a high frequency of SNV mutations in gastrointestinal tumors, as shown in **Figure 1A**. Talin, a major component of the adhesion spot, is responsible for mediating the link between the extracellular matrix and the actin cytoskeleton through integrins. Previous studies have shown that overexpression of talin-1 is associated with increased invasion and reduced survival in oral squamous cell carcinoma, as well as migration, invasion, and apoptosis resistance in prostate cancer cells. Loss of talin-1 results in reduced *in vivo* metastasis of prostate cancer cells *via* the FAK-Src complex and AKT kinase signaling. Talin-1 knockdown also showed a significant reduction in the proliferation, migration, and invasion of CRC cell lines. We also analyzed the copy number variation of ER stress factors in gastrointestinal tumors. The results showed that DNAJB11, EIF4A2, and SERP1 had copy number increases in ESCC, whereas EXOSC10, ZBTB17, and EXTL1 showed copy number loss in LIHC and rectal cancer, as shown in **Figure 1B**.

### Epigenetic changes of ER stressors in digestive system tumors

**Figure 3:**
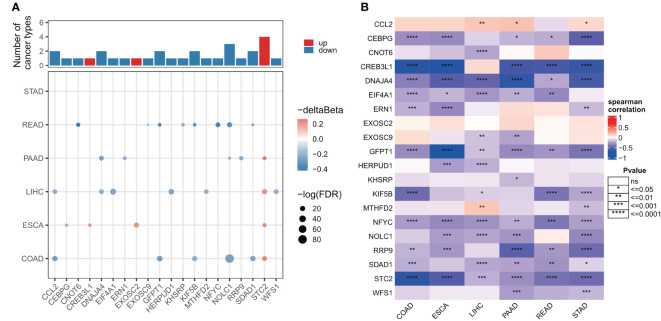
Genomic alterations of endoplasmic reticulum stress factors in gastrointestinal tumors. **(A)** Differential methylation levels of ER stress regulator promoters; **(B)** Correlation between promoter methylation level and mRNA expression of ER stress factors. ns, not significant.

As DNA methylation regulates gene expression in cancer, we next examined the promoter DNA methylation patterns of ER stress factors in digestive system tumors. The results showed that in gastrointestinal tumors, ER stress factors showed less dysmethylation than in para-cancerous tissues (153 ER stress factors; only the genes in **Figure 3A** showed dysmethylation) (**Table S8**). However, these dysmethylated ER stress agents mainly showed hypomethylation levels, among which, NOLC1 showed hypomethylation in PAAD, COAD, and READ, while STC2 showed hypermethylation in LIHC, ESCA, COAD, and PAAD (**Figure 3A**). We also observed an overall inverse correlation between DNA methylation and ER stress factor expression in digestive tract tumors (**Figure 3B**). These results suggest that promoter DNA methylation regulates ER stress factor expression in digestive system tumors.

### Effects of ER stress factors on carcinogenesis pathways

**Figure 4:**
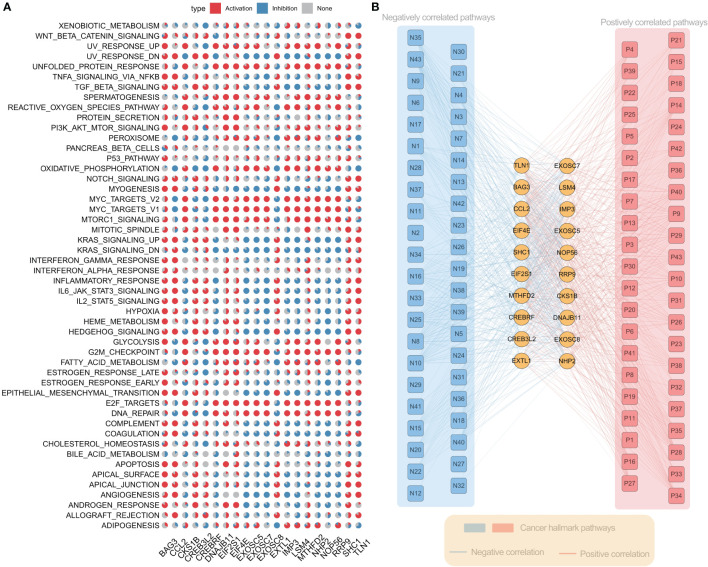
Er stress factors are associated with the activation or inhibition of oncogenic pathways. **(A)** The correlation between endoplasmic reticulum stress factor and Hallmark pathway in gastrointestinal pan-cancer. Red indicates positive correlation, while blue indicates negative correlation. The proportion of pie chart indicates the number of cancers. **(B)** Correlation between ER stress factors and Hallmark pathway in LIHC, with red representing positive correlation and blue representing negative correlation.

To further understand the molecular mechanism of ER stress factors in tumorigenesis, we calculated the Hallmark pathway score based on the ssGSEA algorithm and calculated the Spearman correlation between the expression of individual ER stress factors and signaling pathways (**Table S9**). The top 20 ER stress factors with the greatest associations with the HallMark pathway were selected for the demonstration. The results showed that ER stress factors were highly correlated with the activation or inhibition of several oncogenic pathways. BAG3 was associated with activation of PI3K_AKT_MTOR_SIGNALING, APOPTOSIS, GLYCOLYSIS, and other signaling pathways, while RRP9 is associated with the inhibition of ANGIOGENESIS, MYOGENESIS, and other signaling pathways (**Figure 4A**). These results suggest that ER stress factors are involved in the alteration of various oncogenic pathways in gastrointestinal system tumors. Additionally, the correlation analysis between ER stress factors and pathways showed that the most significant correlation pairs were found in LIHC. Therefore, the correlation analysis results of LIHC were selected for further demonstration (**Figure 4B**).

**Figure 5:**
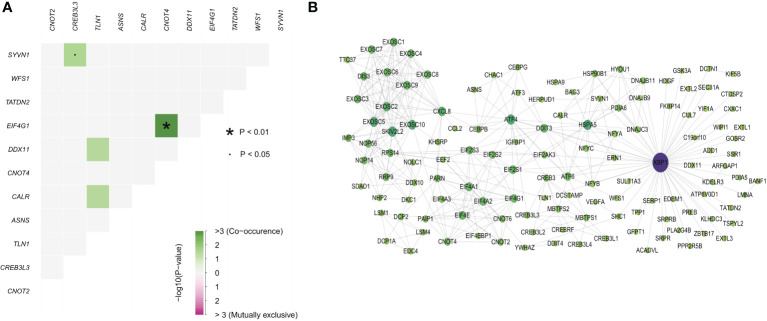
Endoplasmic reticulum stress factor comutations and protein interaction networks. **(A)** Comutation of ER stress factor with SNV mutation frequency greater than 1% in LIHC; **(B)** Protein interaction network of ER stress factors.

As different genes showed interactions, we next investigated genetic alterations and protein-protein interaction networks between ER stress factors. The ER stress factors with an SNV mutation frequency > 1% in LIHC were selected for computation analysis (**Figure 5A**). The results showed that EIF4G1 and CNOT4, CREB3L3, and SYVN1 had obvious co-mutations. The protein interaction network showed that the XBP1 gene had more degrees in the network, suggesting that the XBP1 gene plays an important role in the ER stress regulatory network (**Figure 5B**).

### ER stress factors predict clinical outcomes in patients

**Figure 6:**
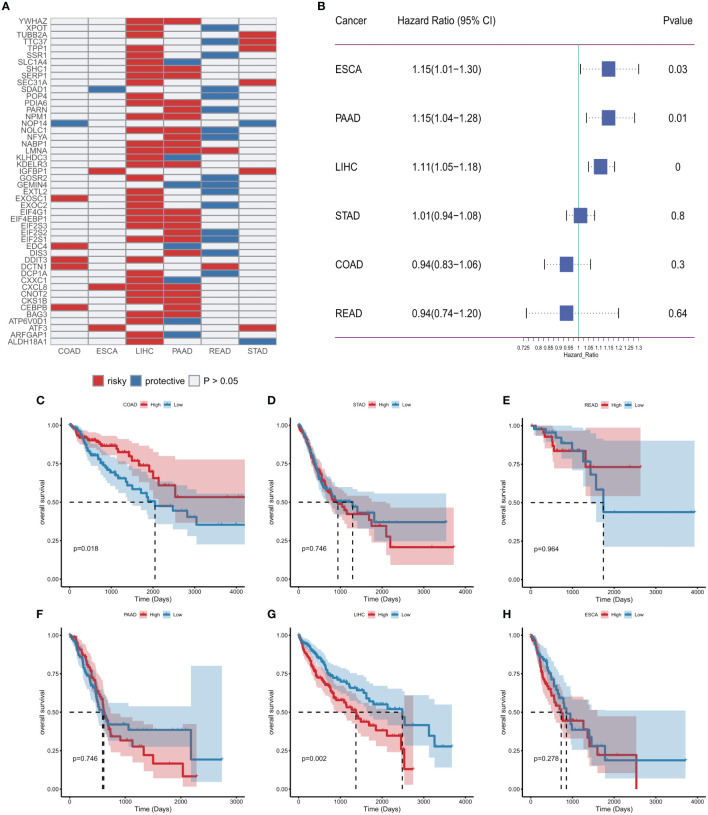
Effect of endoplasmic reticulum stress factors on prognosis. **(A)** Endoplasmic reticulum stress factors associated with overall survival in at least two cancers; **(B)** Results of univariate Cox analysis of CXCL8; **(C–H)** KM curves of CXCL8 in different tumors (median group).

Considering the important association of ER stress factors with cancer, we next evaluated the prognostic efficacy of ER stress factors in gastrointestinal tumors (**Table S10**). Univariate Cox analysis was performed on 153 ER stress factors in different digestive tract tumors, selecting for presentation ER stress regulators associated with overall survival in at least two cancers. The results showed that 48 ER stress factors were associated with overall survival in various digestive tract tumors, and all were risk factors in various cancers, as shown in **Figure 6A**. The effect of ER stress factors on overall survival in rectal adenocarcinoma was the opposite to that in other digestive tract tumors, suggesting that ER stress factors have differential potential for prognostic stratification and development of novel therapeutic strategies for specific types of cancer. The forest plot shows the univariate results of CXCL8 in different gastrointestinal tumors (**Figure 6B**), and the KM curve shows that the effect of CXCL8 on prognosis in different gastrointestinal tumors (**Figures 6C–H**).

### Potential therapeutic effects of ER stressors

**Figure 7:**
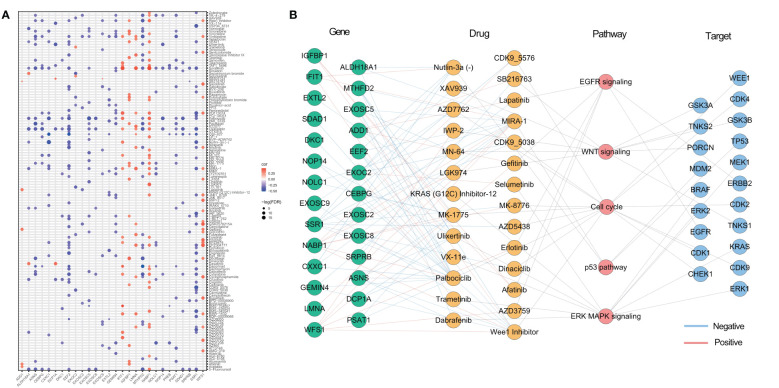
Potential therapeutic effects of ER stress factors. **(A)** The correlation between drug sensitivity and autophagy regulatory factors, and the demonstrated autophagy regulatory factors were correlated with at least five drugs; **(B)** Drugs targeting EGFR, WNT, p53, Cell cycl, ERK MAPK signaling pathways, the targets of these drugs, and the endoplasmic reticulum stress factors related to these drugs.

As identified in the Cancer Drug Sensitivity Genomics (GDSC) project, many clinically actionable genes are targets for anticancer drugs. In order to further assess the potential impact of ER stress factors on drug response, we analyzed the Spearman correlations between the drug sensitivity of 179 gastrointestinal anticancer drugs and transcriptional expression of 153 ER stress factors in 769 cancer cell lines of the GDSC2 cohort (**Table S11**). Among them, 28 genes were correlated with at least five anticancer drugs (**Figure 7A**). For anticancer drugs, ER stress factors were highly correlated with drugs targeting pathways such as EGFR, p53, and WNT. **Figure 7B** shows the hierarchical regulatory network of ER stress factor-drug, pathway-target targeting these important metabolic pathways.

### Effects of ER stress factors on the TME

**Figure 8:**
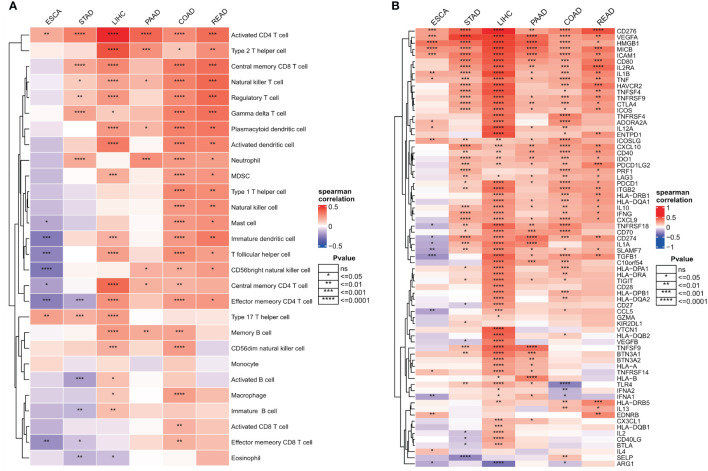
Relationship between endoplasmic reticulum stress scores and tumor microenvironment. **(A)** Spearman’s correlation heat map between endoplasmic reticulum stress scores and immune cell infiltration scores; **(B)** Spearman correlation heat map between ER stress scores and immunomodulators. ns, not significant.

Invasive immune cells are an integral component of the TME and play an important role in improving the effectiveness of immunotherapy. To further evaluate the correlation between the ER stress score and immune cell infiltration, we quantified the immune cell infiltration score in the TME using the ssGSEA method, and the correlation between ER stress score and immune cell infiltration was calculated by Spearman correlation. The results showed that ER stress scores were significantly positively correlated with increased immune cell infiltration in digestive system tumors (**Figure 8A**). To better understand the molecular link between ER stress scores and tumor immunity, we calculated Spearman correlations between ER stress scores and immunomodulators that are critical in immunotherapy. Consistent with the results of our pathway analysis, the ER stress scores were mainly positively correlated with immune modulators in various cancers (**Figure 8B**).

### Cellular sources of dysregulated ER stress factors

**Figure 9:**
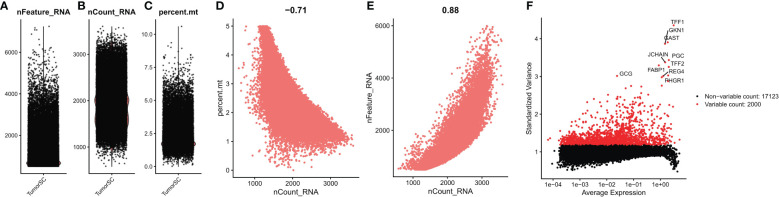
Quality control of single cell data. **(A)** represents the number of genes detected per cell; **(B)** represents the sum of the expression of all genes measured in each cell; **(C)** the proportion of mitochondrial genes detected; **(D)** Correlation between nFeature_RNA and percent.mt; **(E)** Correlation between nCount_RNA and nFeature; **(F)** Screening the top 2000 highly variable genes.

We first performed quality control analysis of the scrNA-SEq dataset GSE134520. The NFeature_RNA (the number of genes detected per cell), nCount_RNA (the sum of all genes detected per cell), and Percent. Mt (the percentage of mitochondrial genes) were calculated. The correlations between nCount_RNA and nFeature, nFeature_RNA, and Percent. Mt were calculated (**Figures 9A–E**). Finally, we selected 200&LT; nFeature_RNA &lt; 6000, and Percent. Mt & lt; 5, and the top 2000 highly variable genes were selected by “FindVariableFeatures” function (**Figure 9F**).

**Figure 10:**
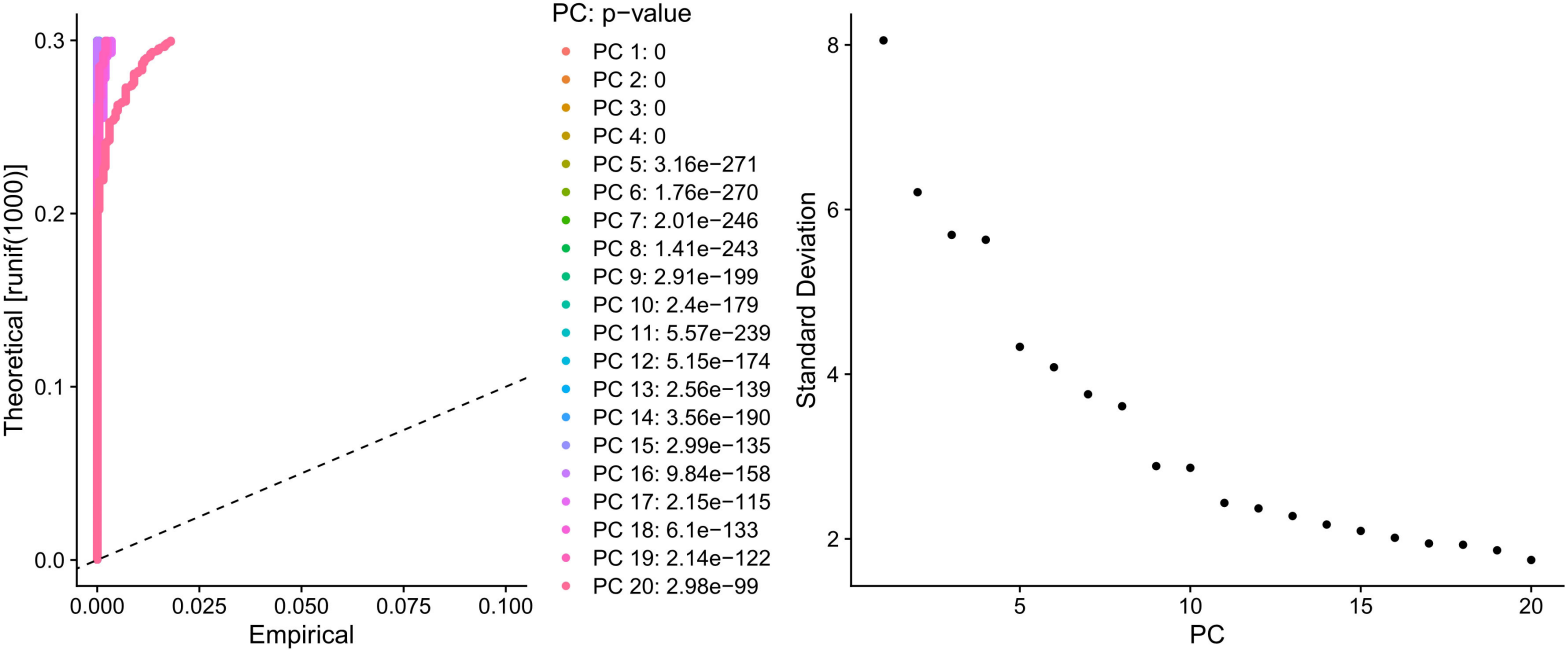
Dimensionality reduction results of PCA for 2000 hypervariable genes.

**Figure 11:**
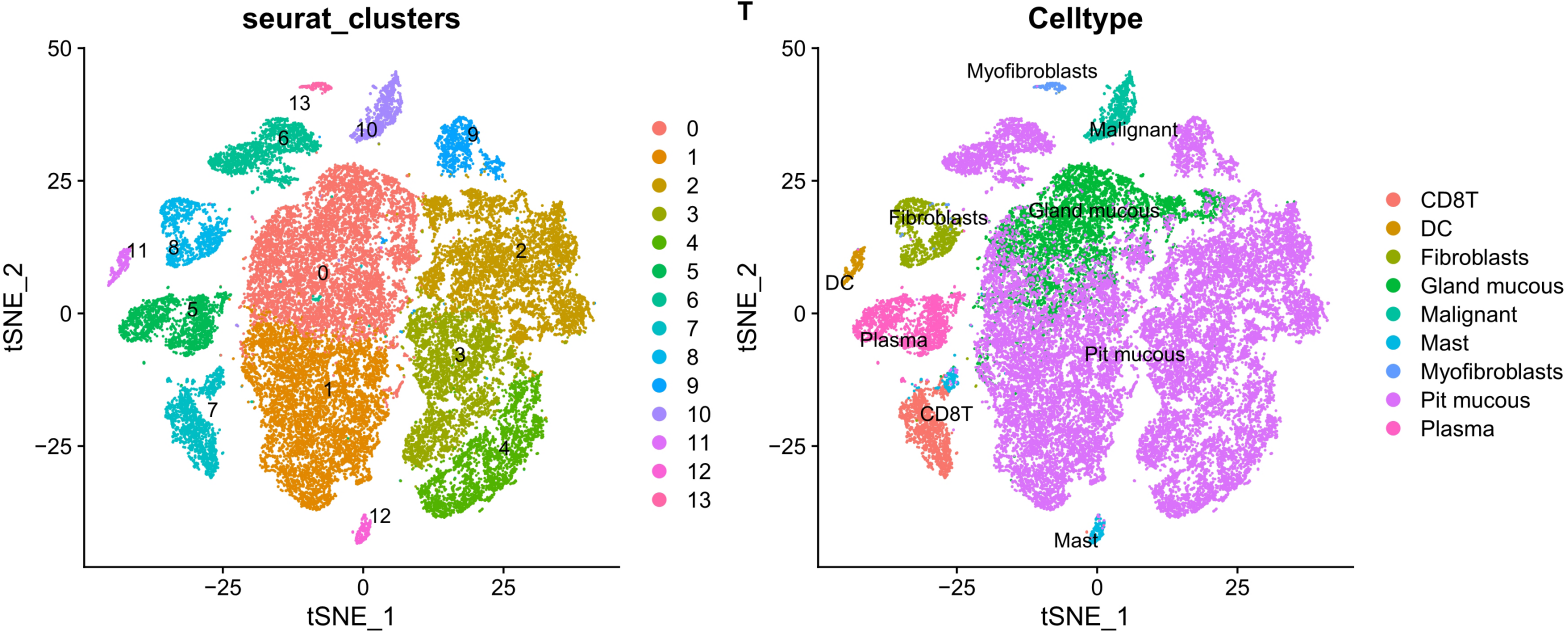
The tSNE algorithm reduced the dimension of 15 PCA and classified 9 kinds of cells.

**Figure 12:**
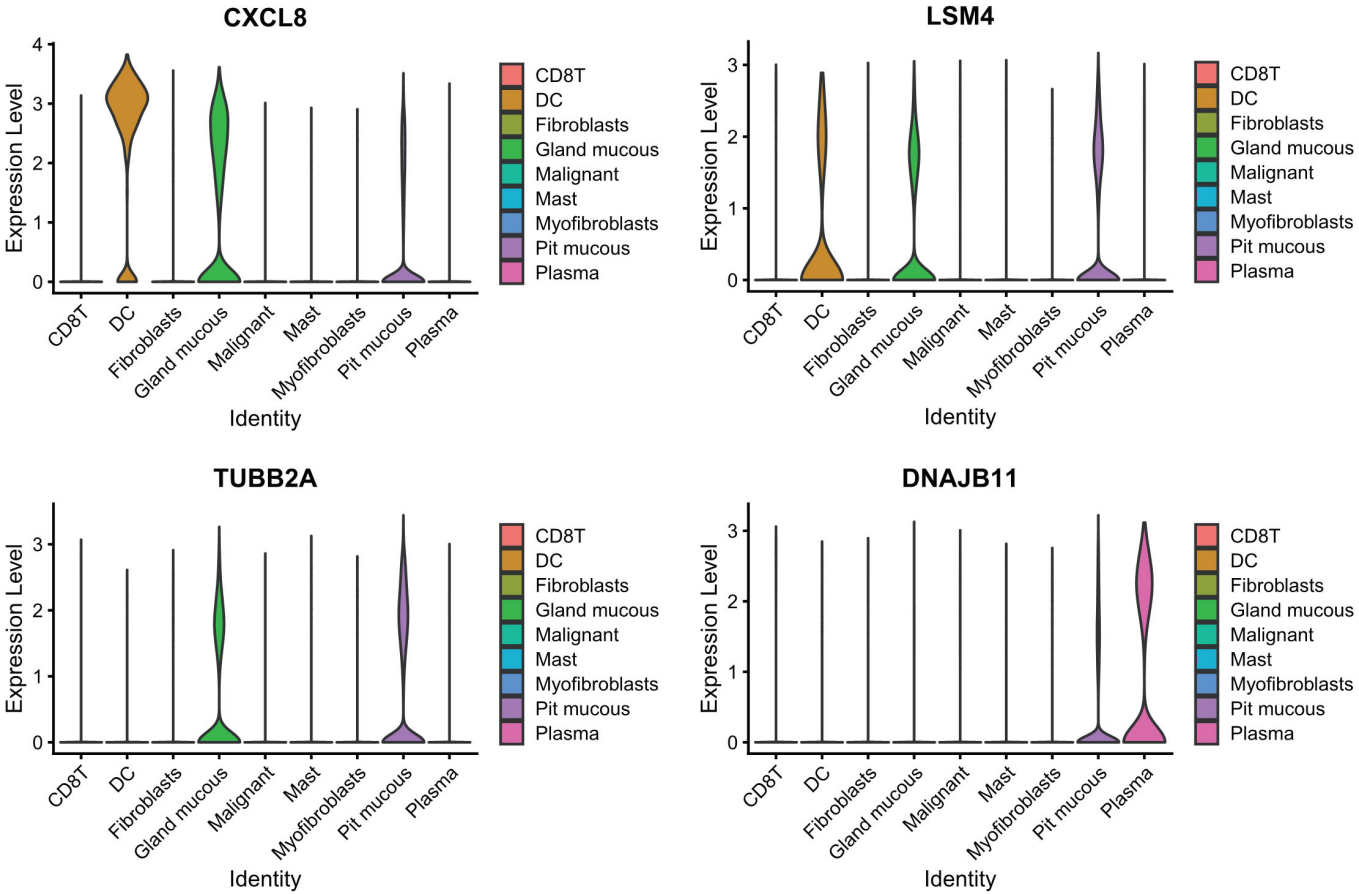
Expression of endoplasmic reticulum stress factors in different cell types.

PCA dimensionality reduction was performed on the 2000 highly variable genes to identify anchors, and the first 15 principal components were selected for subsequent analysis (**Figure 10**). Next, the tSNE algorithm was applied to successfully divide all cells into nine independent types, (**Figure 11**). Finally, the violin plot in **Figure 12** shows the expression of some ER stress factors in different cell types; the single-cell data results for STAD are shown here, while the single-cell numbers for other cancers are shown in the attachment.

## Conclusion

Although ER stress in tumors has been extensively explored (20–22), few studies examined the relationship between ER stress and gastrointestinal tumors. Tumor cells are often affected by internal and external factors, which alter protein homeostasis and cause ER stress. As an reaction, cells invoke an adaptive mechanism to restore protein balance in the ER, namely, the UPR (23, 24). Most UPR signaling pathways are initiated by IRE1α, PERK, and ATF6, making them critical for tumor growth and aggressiveness, microenvironmental remodeling, as well as treatment resistance (25, 26). Persistent ER stress, an emerging hallmark of cancer, is driven by multiple metabolic and oncogenic abnormalities in the TME that perturb protein folding homeostasis in malignant and infiltrating immune cells (1). A constitutively active ER stress response enables malignant cells to adapt to oncogenic and environmental challenges while coordinating multiple immune regulatory mechanisms that promote malignant progression (27). A systematic deconvolution of the precise effects of the UPR on individual cell types in the TME, especially on the metabolic reprogramming of cancer cells *in vivo*, should be performed in the future (28). Although multiple studies have elucidated the function and mechanism of the UPR at every step of tumorigenesis, the role of ER stress in cancer metastasis and resistance to therapy remain poorly understood. In particular, a deeper mechanistic understanding of the UPR in the rate-limiting step of the metastasis cascade, especially in the context of TME reprogramming during metastasis, is important for the rational design of effective therapeutic interventions to address current clinical challenges and improve patient outcomes.

In addition to immune cells, endothelial cells and CAF are important components of the TME and contribute significantly to tumor progression (29, 30). Despite limited knowledge of the role of ER stress in these cells in the TME, there is evidence for the functional effects of ER stress responses on endothelial cells under normal physiological conditions (31, 32). For example, BiP relocalizes from the ER to the cell surface and interacts with T-cadherin to promote endothelial cell survival through the PI3K-Akt pathway (33). Further studies are needed to dissect the role of UPR in endothelial cells, CAF, and other stromal cells in the TME during cancer progression and treatment.

Although targeting the ER stress response alone can disrupt certain aggressive features of cancer cells while enhancing antitumor immunity, this may not result in a therapeutic effect superior to standard interventions (34). To the contrary, there is increasing evidence that modulating ER stress sensors or factors related to the UPR improves susceptibility of aggressive tumors to cytotoxic agents, targeted therapies, and immunotherapies. Larger preclinical studies that recapitulate human tumor heterogeneity using PDXs and immunocompetent mouse models, as well as retrospective analyses of clinical trial samples, could help to identify effective UPR-targeted combination therapies to elicit durable responses that prevent cancer progression and/or recurrence.

Here we used the limma package to analyze the differences in ER stress factors among pan-cancers in the digestive tract and uncover the positively correlated ER stress factors and digestive tract tumors. This is supported by the DDX11, DKC1, EXOSC5, NOP56, and other genes showing differences in the most cancer types. To investigate genomic alterations in ER stress factors in digestive tract tumors, we calculated the frequencies of CNV variants (amplifiers and deletions) and SNV mutations (non-silent mutations) in pan-cancer cohorts of patients with six cancer types. We next observed an overall inverse correlation between DNA methylation and ER stress factor expression in digestive tract tumors. These results suggest that promoter DNA methylation regulates the expression of ER stress factors in digestive system tumors. The Hallmark pathway score determined by the ssGSEA algorithm, and the genetic changes and protein interaction network results showed that ER stress factors are highly associated with the activation or inhibition of multiple oncogenic pathways. The ssGSEA method was used to quantify the score of immune cell infiltration in the TME, and Spearman correlation was used to calculate the correlation between the ER stress score and immune cell infiltration, which was consistent with the results of pathway analysis. The score of ER stress and immune modulators were mainly positively correlated in many cancers. To identify the cellular origin of ER stress factor dysregulation, we targeted scrNA-seq analysis and selected 2000 highly variable genes. Subsequently, PCA was used to reduce the dimensions of 2000 genes to identify the anchor points, 15 principal components were selected, and the tSNE algorithm was used to obtain nine independent cell types. Considering the important association between ER stress factors with cancer, we evaluated the prognostic efficacy of ER stress factors in gastrointestinal tumors. The effect of ER stress factors on overall survival in rectal adenocarcinoma was opposite to that in other digestive tract tumors, suggesting that ER stress factors have differential potential for the prognostic stratification and development of new therapeutic strategies for specific types of cancer. To further assess the potential effect of ER stress factors on drug response, we analyzed the Spearman correlations between the drug sensitivity of 179 gastrointestinal anticancer drugs and the transcriptional expression of 153 ER stress factors in 769 cancer cell lines from the GDSC2 cohort.

our results cannot exclude the possibility that other key clinical factors may influence the patient outcome, because first our study lacks external validation, and second, the clinical data of some important parameters were incomplete due to database limitations, which may affect the statistical power. Nevertheless, the value of ER stress factors in digestive tract tumors needs to be verified by more trials.

## Data availability statement

The transcriptional data, mutation data, methylation data, and survival information provided in the study are deposited in the TCGA database (https://portal.gdc.cancer.gov/), accession numbers: TCGA-COAD, TCGA-ESCA, TCGA-LIHC, TCGA-PAAD, TCGA-READ, TCGA-STAD. Data on normal digestive tract tissues provided in the study are deposited in the GTEx database at the following links: Colon-Sigmoid, Colon–Transverse, Esophagus-Gastroesophageal Junction, Liver, Pancreas, Stomach. The oncopathway presented in the study are deposited in the Hallmark gene sets of the MSigDB database. The drug sensitivity data in the study are deposited in the GDSC2 dataset of the GDSC database (https://www.cancerrxgene.org/). The gastric cancer single cell data used in the study are deposited in the GEO database (https://www.ncbi.nlm.nih.gov/geo/), accession number GSE134520.

## Author contributions

SY performed all the experiments and XP wrote the manuscript with contributions from all authors. YY and LX is responsible for language polishing and double-checking all data. All authors contributed to the article and approved the submitted version.
