# Supplementary figures and images for: Genomic and clinical features of endoplasmic reticulum stress factor in digestive system pan-cancer studies
Source: Front Oncol. 2023 Jan 9;12:1072576. doi: 10.3389/fonc.2022.1072576 (PMC9868864; doi:10.3389/fonc.2022.1072576)

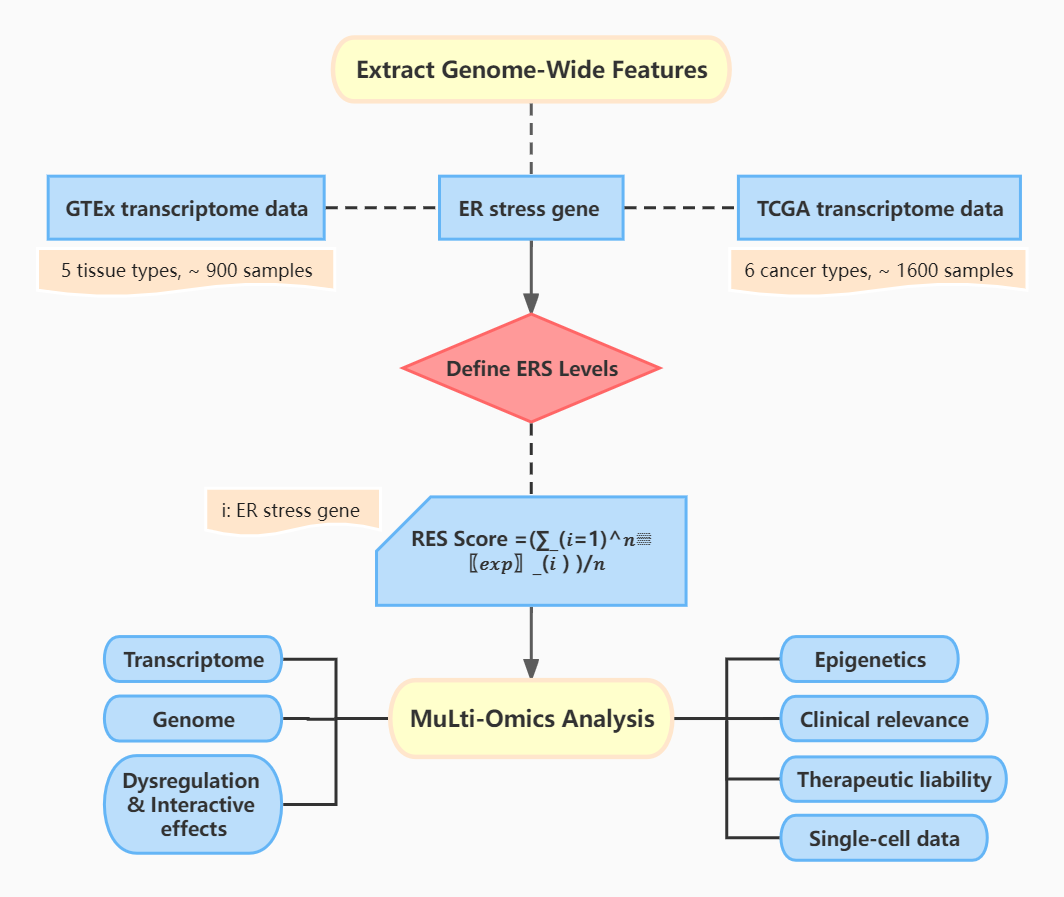

Supplement: Supplementary file 12 [file Image_1.jpeg]
